# Supplementary material for: tRNA-derived RNA processing in sperm transmits non-genetically inherited phenotypes to offspring in C. elegans
Source: Res Sq. 2025 Jun 6:rs.3.rs-6770943. Preprint. [Version 1] doi: 10.21203/rs.3.rs-6770943/v1 (PMC12155203; doi:10.21203/rs.3.rs-6770943/v1)
Supplement: 1 [file NIHPPRS6770943V1-supplement-1.pdf]

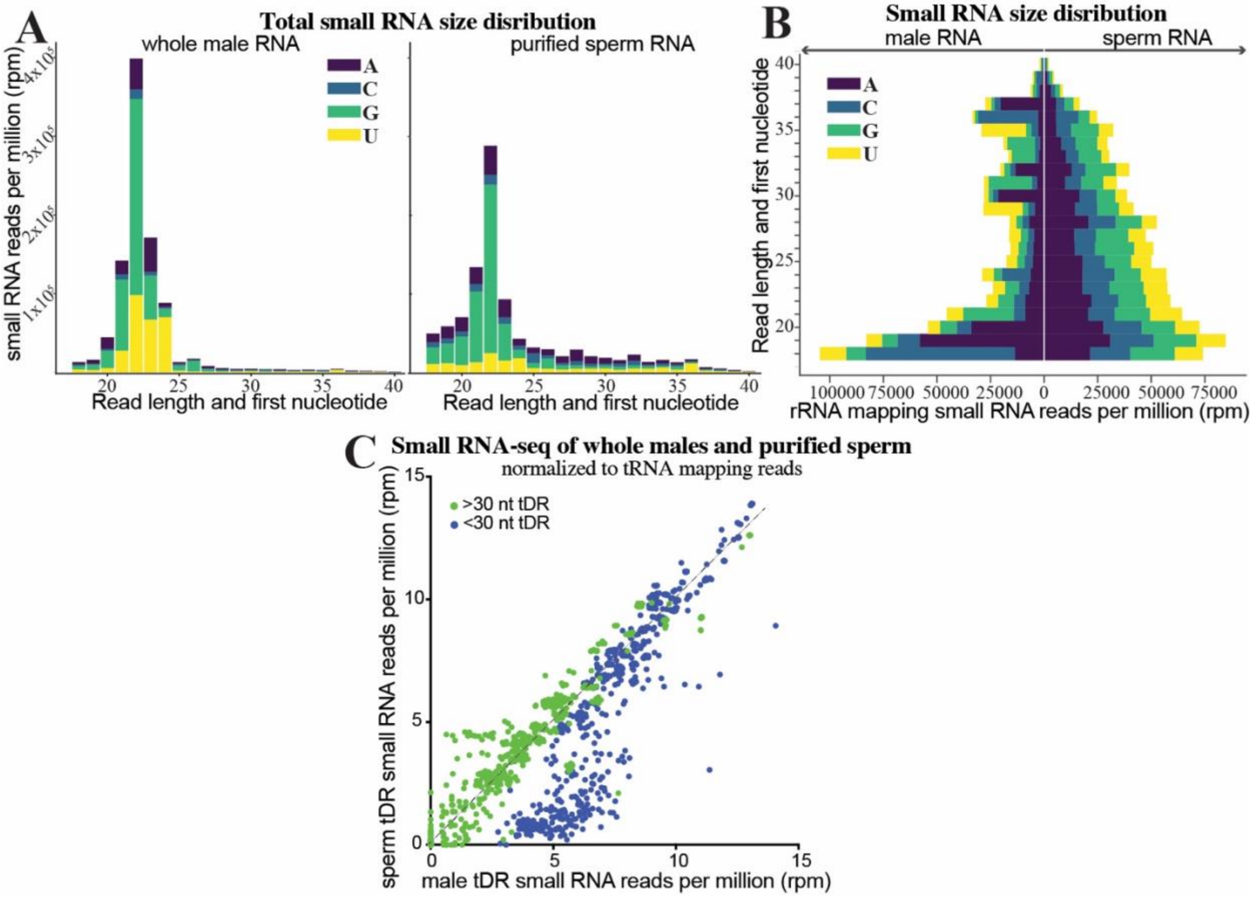

Supplemental Figure 1. Small RNA-seq reveals that tDRs are enriched in *C. elegans* sperm. Small RNA-seq was performed on PAGE purified 18-40 nt RNA from *C. elegans* whole males and purified sperm. **(A)** Length (x-axis) and first nucleotide distribution (legend) of all genome mapping reads. Whole male data (left) and purified sperm (right). **(B**) Reads mapping to rRNAs were quantitated for RNA length (y-axis) and plotted as their starting nucleotide in a diverging bar-graph (x-axis – total quantitated reads). **(C)** tRNA mapping reads were separated as >30 or <30 nucleotides in length and quantitated, as reads per million (rpm) tRNA mapping reads, for all tRNA isoacceptor genes. Each dot represents a quantitated tRNA isoacceptor (green >30 and blue <30 nucleotide tRNA mapping reads per tDR).

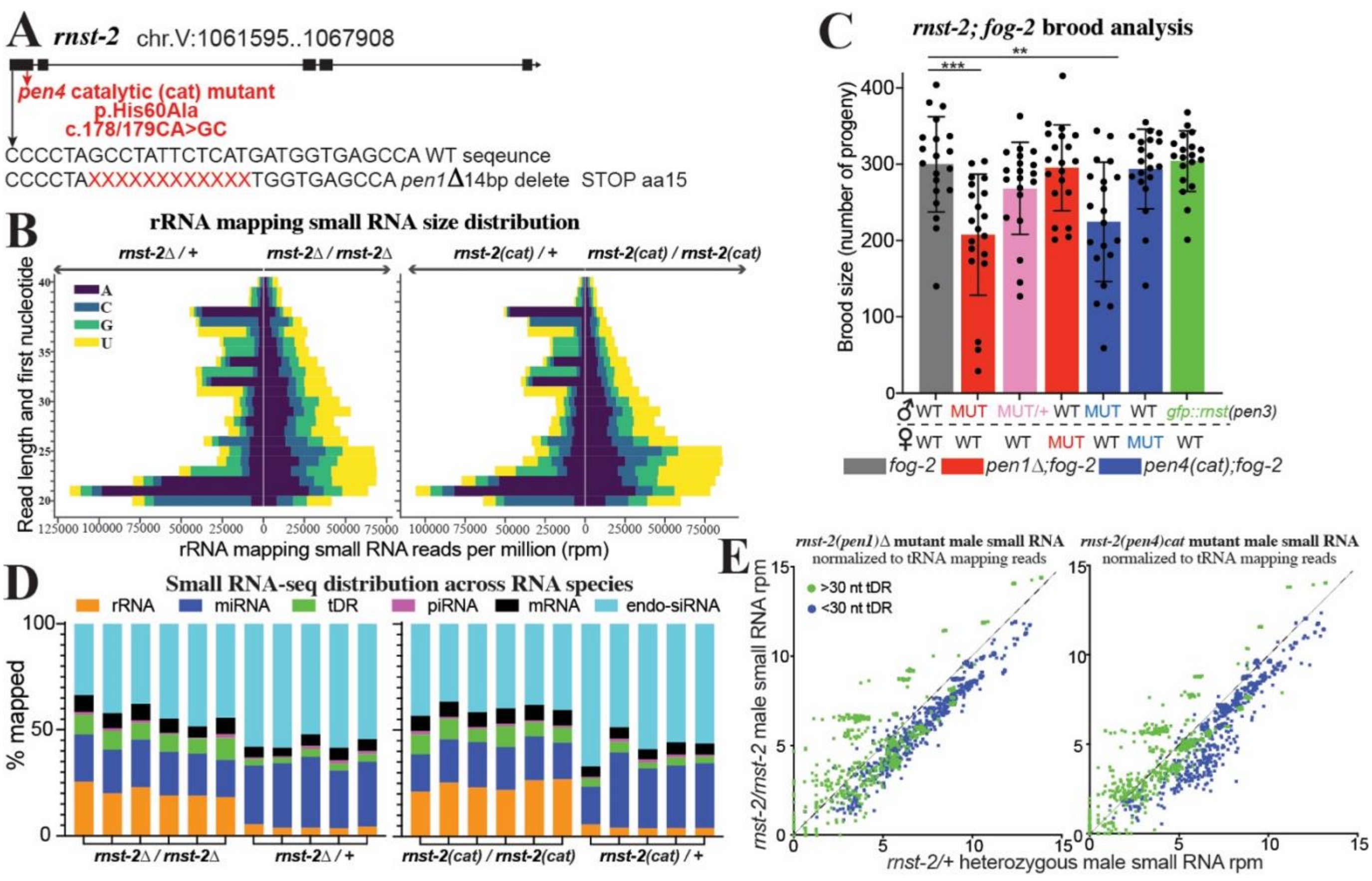

Supplemental Figure 2. *rnst-2* regulates tDR accumulation and length. Small RNA-seq was performed on *C. elegans* whole male *rnst-2* mutant (*pen1* – Δ and *pen4* – catalytic) as well as heterozygous controls. **(A)** Schematic of the *rnst-2* locus. Additionally, mutant alleles generated in this study in the proceeding experiments are annotated. **(B)** Reads mapping to rRNAs were quantitated for RNA length (y-axis) and plotted as their starting nucleotide in a diverging bar-graph (x-axis – total quantitated reads). **(C)** Fecundity was measured by assessing the brood size (# of progeny generated) of *rnst-2*$^{\Delta/\Delta}$, *rnst-2*$^{cat/cat}$, heterozygous controls (*rnst-2*$^{\Delta/+}$ & *rnst-2*$^{cat/+}$), endogenously(CRISPR) tagged *gfp::rnst-2*, and WT (*fog-2*) males mated with WT *fog-2* females (**$p_{val} < 0.01$, ***$p_{val} < 0.001$ – two-sample unpaired t-test). Additionally, fecundity of WT *fog-2* males mated with *rnst-2* mutant females was assessed. **(D)** Total small RNA reads mapping to each class of RNAs quantitated as percentage of total genome mapping reads as stacked bar graphs for each replicate of the experiment (n = 4-5). **(E)** For each mutant and their respective heterozygous control, tRNA mapping reads were separated as >30 or <30 nucleotides in length and quantitated, as reads per million (rpm), for all tRNA isoacceptor genes. Each dot represents a quantitated tRNA isodecoder (green >30 and blue <30 nucleotide tRNA mapping reads for each isodecoder).

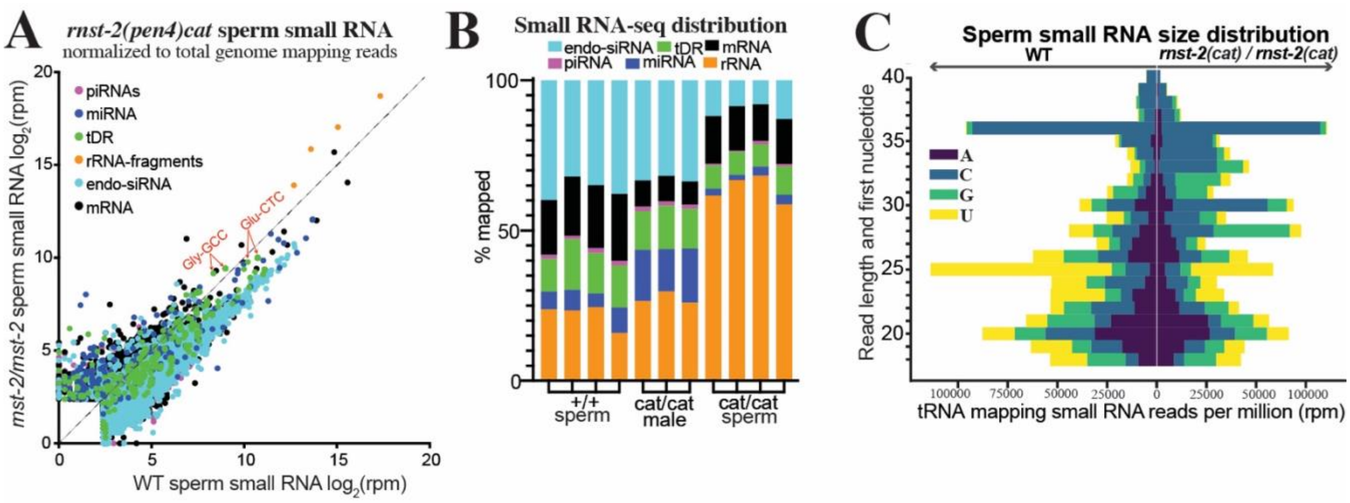

Supplemental Figure 3. ***rnst-2*** **regulates tDR accumulation in sperm.** Small RNA-seq was performed on *rnst-2(cat)* sperm and whole males and compared to WT sperm small RNA data. **(A)** Scatter plot of small RNA-seq data normalized to total genome mapping reads from WT sperm (x-axis – also Fig.1) and *rnst-2*$^{cat/cat}$ mutant sperm (y-axis). **(B)** Distribution of small RNAs mapped to all small RNA classes from WT and *rnst-2*$^{cat/cat}$ mutant sperm and males. Each biological replicate from the experiment is represented as an individual bar graph. **(C)** Reads mapping to tRNAs were quantitated for RNA length (y-axis) and plotted as their starting nucleotide (legend) in a diverging bar-graph (x-axis – total quantitated reads) for WT and *rnst-2*$^{cat/cat}$ mutant sperm.

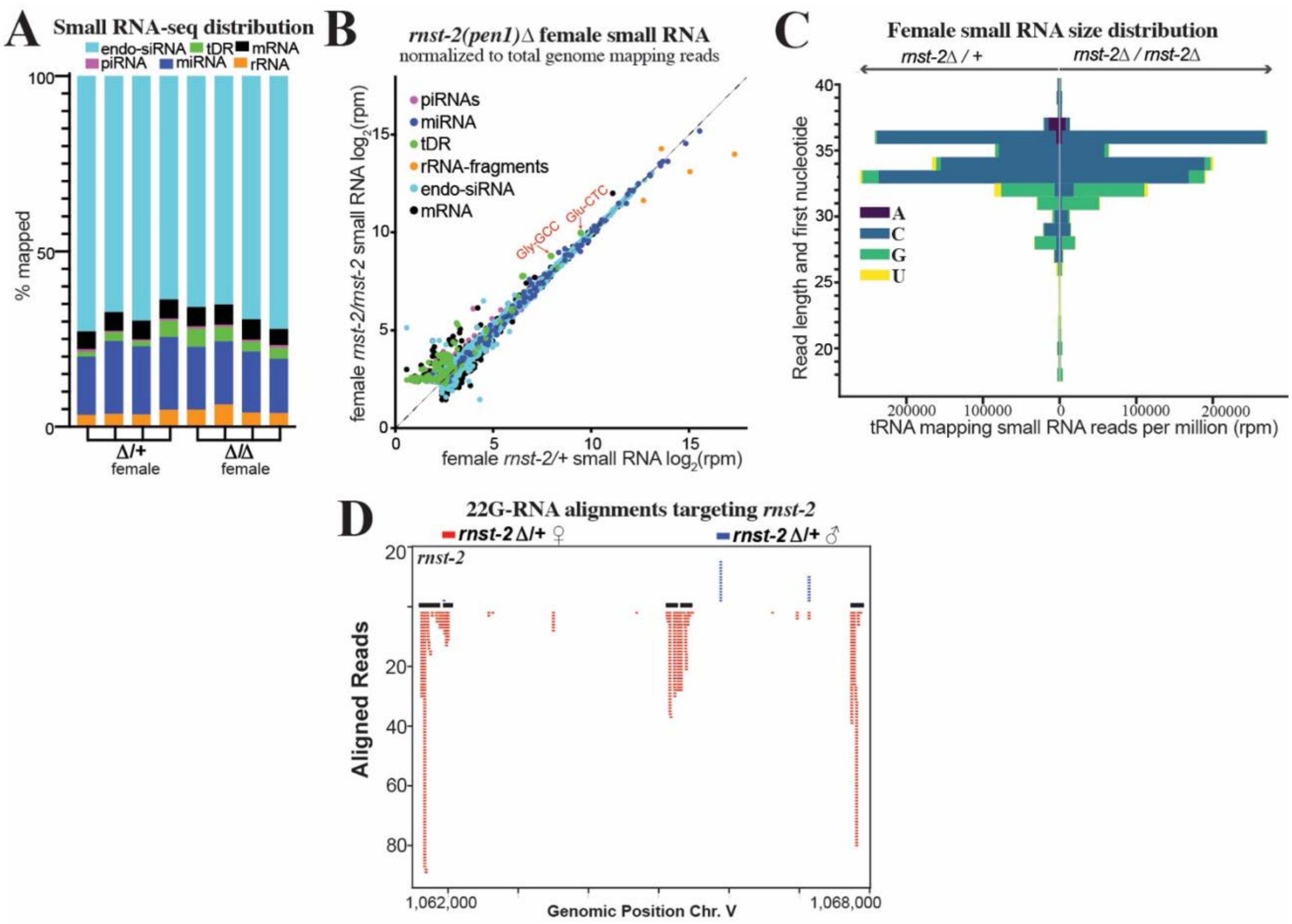

Supplemental Figure 4. *rnst-2* dependent regulation of tDRs is suppressed in females. Small RNA-seq was performed on *C. elegans* whole female (without embryos) *rnst-2*$^{\Delta/\Delta}$ mutants as well as heterozygous controls. **(A)** Distribution of small RNAs mapped to all small RNA classes from *rnst-2*$^{\Delta/+}$and *rnst-2*$^{\Delta/\Delta}$ females. Each biological replicate from the experiment is represented as an individual bar graph (n=4). **(B)** Scatter plot of small RNA-seq data normalized to total genome mapping reads as reads per million (rpm) from *rnst-2*$^{\Delta/+}$ (x-axis) and *rnst-2*$^{\Delta/\Delta}$ females (y-axis). **(C)** Reads mapping to tRNAs were quantitated for RNA length (y-axis) and plotted as their starting nucleotide (legend) in a diverging bar-graph (x-axis – total quantitated reads) for *rnst-2*$^{\Delta/+}$and *rnst-2*$^{\Delta/\Delta}$ females. **(D)** 22G endo-siRNA reads aligned to the *rnst-2* gene locus in *rnst-2*$^{\Delta/+}$ male (Fig. 2) and female small RNA data.

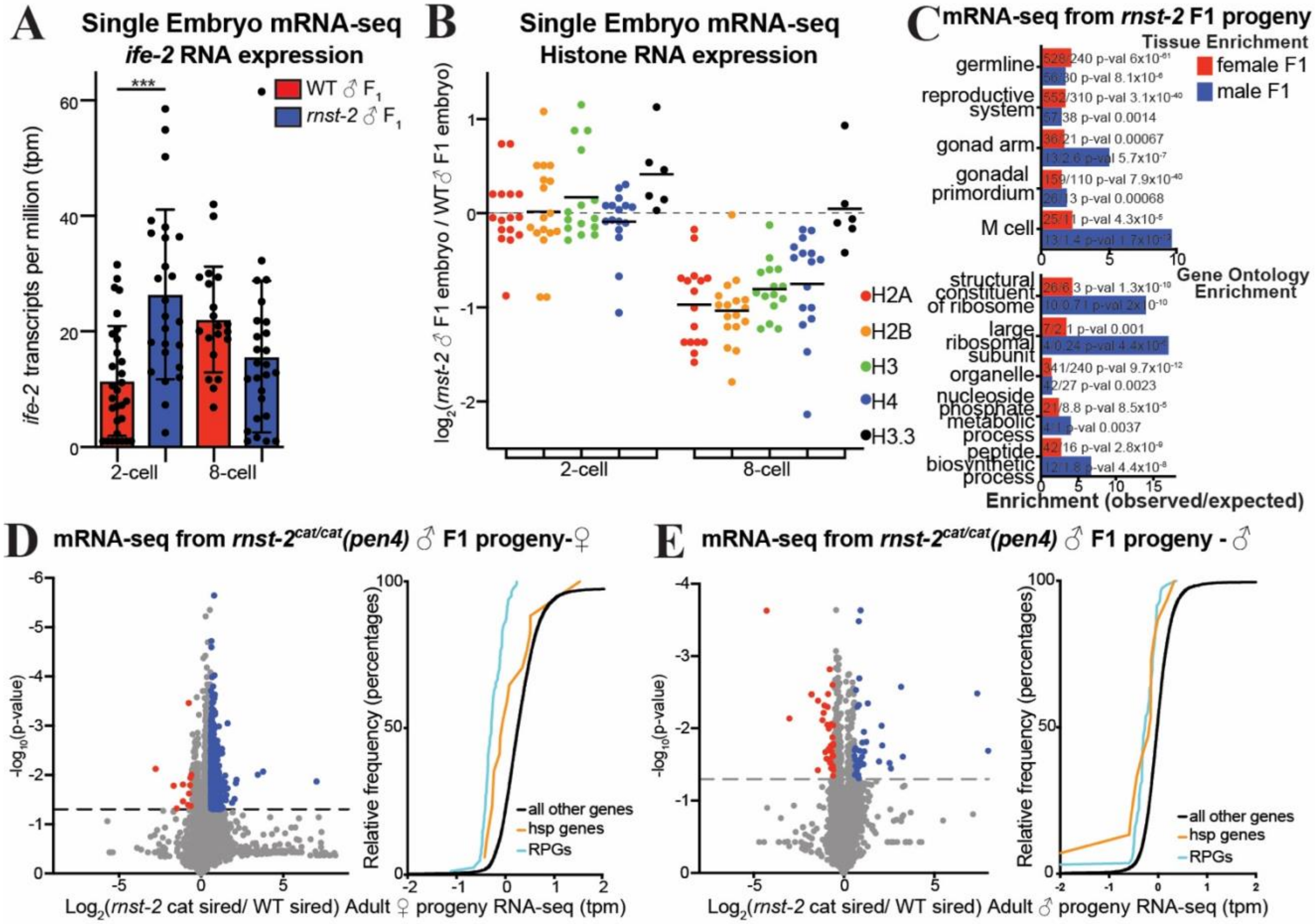

Supplemental Figure 5. ***rnst-2*** **mutant males transmit non-genetically inherited phenotypes to offspring. (A)** Bar-plot representing *ife-2* expression in transcripts per million (tpm) for each replicate in our dataset in 2- and 8-cell embryos sired by *rnst-2*$^{\Delta/\Delta}$ and WT males (***$p_{val < 0.001}$ – two-sample t-test); n = 20-30. **(B)** Expression of histone RNAs grouped by type of histone gene in 2- and 8-cell embryos sired by *rnst-2*$^{\Delta/\Delta}$ and WT males. Each dot represents the average expression of a specific histone gene in *rnst-2*$^{\Delta/\Delta}$ relative to WT sired progeny. **(C)** Significantly regulated Gene Ontology and Tissue Enrichment terms for genes differentially expressed in mRNA-seq data from male and female progeny of *rnst-2*$^{\Delta/\Delta}$ males. **(D-E)** Volcano plots graphing the mRNA-seq data of progeny of *rnst-2*$^{\Delta/\Delta}$ males compared to WT male progeny. Additionally, cumulative distribution frequency plots of gene expression of *rnst-2*$^{\Delta/\Delta}$ relative to control progeny for all genes, heat-shock genes, and ribosomal protein genes (RPGs). **(D)** Female progeny mRNA-seq. **(E)** Male progeny mRNA-seq.

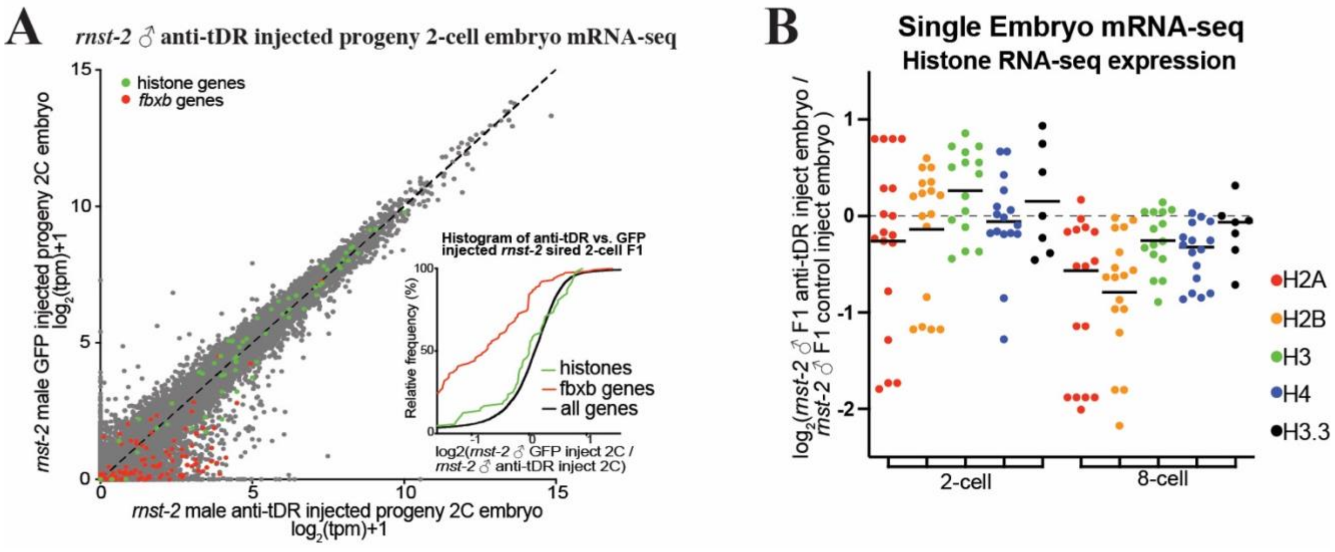

Supplemental Figure 6. tDRs causally transmit non-genetically inherited phenotypes to progeny. (A-B) Single-embryo mRNA-seq was performed on 2-cell and 8-cell embryos sired by *rnst-2*$^{\Delta/\Delta}$ males mated with WT females and then microinjected with either anti-tDR (anti-Gly-GCC or anti-Glu-CTC) + GFP mRNA or GFP mRNA alone as a microinjection control. **(A)** tpm normalized mRNA-seq data represented as a scatter plot of 2-cell embryo progeny of *rnst-2*$^{\Delta/\Delta}$ males mated with WT females injected with anti-tDR (x-axis) versus 2-cell embryo progeny of *rnst-2*$^{\Delta/\Delta}$ mated with WT female injected with GFP mRNA (y-axis). The inset graph represents cumulative distribution frequency plots of all genes, histone genes (colored green), and *fbxb* genes (colored red). n = 26 GFP injected, n = 23 anti-tDR injected **(B)** Expression of histone RNAs grouped by type of histone gene in 2 and 8-cell embryos sired by *rnst-2*$^{\Delta/\Delta}$ males with WT females microinjected with either anti-tDR or GFP control RNA. Each dot represents the average of a specific histone gene's expression in *rnst-2*$^{\Delta/\Delta}$ relative to WT sired progeny.
